# Shortening of atrioventricular delay at increased atrial paced heart rates improves diastolic filling and functional class in patients with biventricular pacing

**DOI:** 10.1186/1476-7120-10-2

**Published:** 2012-01-24

**Authors:** Reza Rafie, Salima Qamruddin, Ali Ozhand, Nima Taha, Tasneem Z Naqvi

**Affiliations:** 1 Echocardiographic Laboratories, Department of Medicine, Keck School of Medicine, University of Southern California, Los Angeles, California, 90033 USA; 2 Department of Preventive Medicine, Keck School of Medicine, University of Southern California, Los Angeles, California, 90033 USA

**Keywords:** Biventricular pacemaker, Rate Adaptive AV Delay, Diastolic Function, Echocardiography, Doppler

## Abstract

**Background:**

Use of rate adaptive atrioventricular (AV) delay remains controversial in patients with biventricular (Biv) pacing. We hypothesized that a shortened AV delay would provide optimal diastolic filling by allowing separation of early and late diastolic filling at increased heart rate (HR) in these patients.

**Methods:**

34 patients (75 ± 11 yrs, 24 M, LVEF 34 ± 12%) with Biv and atrial pacing had optimal AV delay determined at baseline HR by Doppler echocardiography. Atrial pacing rate was then increased in 10 bpm increments to a maximum of 90 bpm. At each atrial pacing HR, optimal AV delay was determined by changing AV delay until best E and A wave separation was seen on mitral inflow pulsed wave (PW) Doppler (defined as increased atrial duration from baseline or prior pacemaker setting with minimal atrial truncation). Left ventricular (LV) systolic ejection time and velocity time integral (VTI) at fixed and optimal AV delay was also tested in 13 patients. Rate adaptive AV delay was then programmed according to the optimal AV delay at the highest HR tested and patients were followed for 1 month to assess change in NYHA class and Quality of Life Score as assessed by Minnesota Living with Heart Failure Questionnaire.

**Results:**

81 AV delays were evaluated at different atrial pacing rates. Optimal AV delay decreased as atrial paced HR increased (201 ms at 60 bpm, 187 ms at 70 bpm, 146 ms at 80 bpm and 123 ms at 90 bpm (ANOVA F-statistic = 15, p = 0.0010). Diastolic filling time (P < 0.001 vs. fixed AV delay), mitral inflow VTI (p < 0.05 vs fixed AV delay) and systolic ejection time (p < 0.02 vs. fixed AV delay) improved by 14%, 5% and 4% respectively at optimal versus fixed AV delay at the same HR. NYHA improved from 2.6 ± 0.7 at baseline to 1.7 ± 0.8 (p < 0.01) 1 month post optimization. Physical component of Quality of Life Score improved from 32 ± 17 at baseline to 25 ± 12 (p < 0.05) at follow up.

**Conclusions:**

Increased heart rate by atrial pacing in patients with Biv pacing causes compromise in diastolic filling time which can be improved by AV delay shortening. Aggressive AV delay shortening was required at heart rates in physiologic range to achieve optimal diastolic filling and was associated with an increase in LV ejection time during optimization. Functional class improved at 1 month post optimization using aggressive AV delay shortening algorithm derived from echo-guidance at the time of Biv pacemaker optimization.

## 

Cardiac resynchronization therapy (CRT) in patients with advanced heart failure leads to an improvement in cardiac function during rest, exercise [1] and pharmacological stress [2]. Previous studies have shown that in patients with CRT, optimizing atrioventricular (AV) delay further improves stroke volume [3-6] and that optimal AV delay changes over time requiring periodic optimization [7]. There is controversy on optimal AV delay at increase in heart rate with exercise. Studies have shown conflicting data suggesting AV delay should be prolonged [8] or shortened [9,10] as heart rate increases. In patients with heart failure, an increase in heart rate can compromise diastolic filling to preserve systolic ejection [11]. This diastolic filling time can be manipulated in heart failure patients with CRT by adjusting AV delay at increased heart rates by using rate adaptive algorithms of CRT devices. In this study in patients with CRT, we evaluated a) the effect of increased atrial pacing rate on diastolic filling at a fixed AV delay and b) to examine the effect of manipulating AV delay on diastolic filling. Our secondary goal was to examine the effect of programming AV delay that provided most optimal diastolic filling at increased heart rate on cardiac ejection acutely and on functional class at 1 month follow up.

## Methods

### Study Subjects

34 heart failure patients with Biv-CRT who were atrially pacing at resting heart rates were included in the study.

### Exclusion criteria

Patients with atrial fibrillation, atrial pacing of less than 50% on pacemaker interrogation, recent hospital admission within 2 months for congestive heart failure and acute coronary event requiring revascularization within 3 months were excluded from the study. All patients signed an informed consent approved by the Institutional Review Board. 14 patients from this study were part of the 2 studies reported earlier [5,6].

### Pacing Protocol

AV delay and interventricular (VV) delays were optimized at rest using Doppler echocardiography by conventional methods. AV delay that provided that most optimal mitral inflow filling without atrial truncation and with best E and A separation was chosen at optimal AV delay. Left ventricular outflow tract (LVOT) pulsed wave (PW) Doppler was measured at rest and at each AV delay along with pulmonary vein PW Doppler. After optimal AV delay was determined at rest, atrial pacing rate was increased at 10 bpm increments from baseline heart rate of 60 or 70 bpm to a maximum of 90 bpm. At each atrial pacing rate, mitral inflow PW Doppler was first evaluated at the AV delay found optimal at the previous paced atrial rate. Then the AV delay was decreased in decrements of 20 ms until best E and A wave separation was seen on mitral inflow without atrial truncation. If E and A separation did not occur despite decreasing AV delay, AV delay was increased in increments of 20 ms until E and A separation was noted. Atrial pacing rate ranged from 60-90 beats per minute. In 13 patients LV ejection time was also evaluated at each paced atrial rate before and after optimal AV delay change. PW Doppler assessment was performed 5 beats of after change in pacemaker setting.

### Echocardiography

We used transthoracic echocardiography using GE Vivid 7 or Vivid 9 ultrasound systems (Horten, Norway) equipped with a variable frequency 2.5-5 MHz transducer with harmonics. Echocardiogram was performed in the left lateral decubitus position. AV delay was first optimized at resting heart rate partially adopting the recent guidelines from American Society of Echocardiography [12]. PW Doppler sample volume placed at the tip of the mitral leaflets for LV diastolic filling parameters. PW Doppler sample volume was placed 0.5-1 cm below the aortic valve at the LV outflow tract (LVOT) to obtain the LV ejection duration and aortic velocity time integral (VTI). Frame rate was kept above 100 fps by using single focus, narrow imaging sector, appropriate depth and frame rate. Parallel Doppler beam alignment to myocardial segments and color Doppler was used for all Doppler data acquisition. ECG was displayed on the ultrasound system and 3 cardiac cycles were used for each data acquisition. Raw data was stored digitally as DICOM cine loops and transferred for offline analysis to a customized dedicated workstation equipped with custom built software (Echo PAC PC Dimension version 6.0.1, GE Vingmed Ultrasound, Horten, Norway) via internet.

### Offline Measurements

All routine chamber dimensions and LV ejection fraction at baseline were measured off line. Mitral inflow peak E and A velocities, E wave deceleration time and mitral inflow VTI were measured at baseline and at each paced heart rate. We also measured LVOT PW Doppler ejection time and VTI in 13 patients at fixed AV delay and optimal AV delay at increased atrial pacing rates. Two blinded independent investigators performed offline analysis of all echo data. Investigators performing offline analysis were blinded to online assessment of optimal AV delay settings.

### Functional Class Assessment

NYHA class was evaluated at baseline and Quality of Life was assessed by Minnesota Living with Heart Failure Questionnaire (MHFQ) at baseline. NYHA class and MHFQ were re-evaluated by a questionnaire given to the patient in a self addressed envelope to be mailed in at 1 month post optimization. A hall walk as tolerated by the patient was performed on all patients after optimization to ensure no deterioration of exercise capacity from baseline.

### Statistical analysis

All measurements were displayed as mean ± SD. Measurements before and after optimization for AV delay for each heart rate as well as NHYA and functional class scores were analyzed by Paired student t- test. For comparison of different heart rates, we used analysis of variance (ANOVA). We used repeated measures ANOVA for optimal measured AV delay at more than two different heart rates. P < 0.05 was considered statistically significant.

## Results

34 patients were included in our study. Baseline characteristics of the study population are shown in Table 1. The mean duration of CRT implant prior to optimization was 23 ± 27 months.

**Table 1:** Baseline Characteristics of the Study Population

Age	75 ± 11 years
**Gender**	Male/Female: 24(72%)/10(28%)

**NYHA**	2.6 ± 0.7 - I/II/III/IV: 3%/34%/47%/16%

**NYHA**	2.6 ± 0.7 - I/II/III/IV: 3%/34%/47%/16%

**Cardiomyopathy**	Ischemic/Non- Ischemic: 69%/31%

**QRS Duration**	175.3 ± 38.6 ms

**Left Ventricular Diastolic Diameter**	5.9 ± 0.93 cm

**Left Ventricular Systolic Diameter**	4.9 ± 1.1 cm

**Left Ventricular Ejection Fraction**	34.1 ± 12.4%

**Left Atrial Diameter (Superior-Inferior)**	6 ± 1 cm

**Left Atrial Diameter (Antero-Posterior)**	4.7 ± 0.8 cm

**AV Delay**	154.1 ± 40.1 (baseline)

### Effect of Optimal AV delay Programming at Resting Paced Atrial Rate

**Figure 1:**
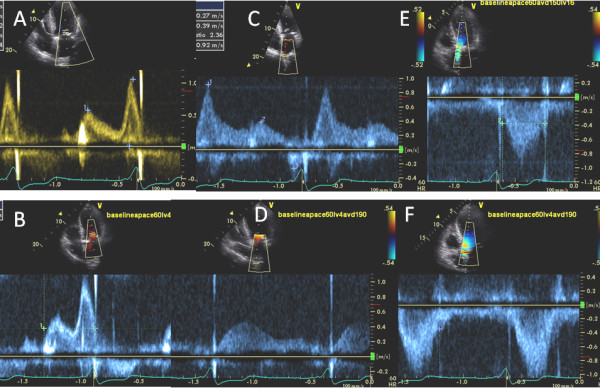
**Effect of AV delay optimization on diastolic filling**. Mitral inflow (A and B), pulmonary vein inflow (C and D), and LV outflow PW Doppler (E and F) at baseline (Apace 60 AV delay 150, LV 16 ms): A, C, and E) and optimal (A-paced 60 beats/min, AV delay 190 ms, VV0 ms: B, D, and F) pacemaker settings. An increase in A wave duration occurred (B), and pulmonary vein pattern changed so that systolic fraction increased, S wave deceleration time increased (D) and increased pulmonary vein atrial reversal (C) was abolished indicating reduction in left atrial pressure. An improvement in percentage LV ejection time also occurred (F).

Change in diastolic filling and systolic ejection PW Doppler parameters and on peak pulmonary artery pressure from baseline AV delay to optimal AV delay at resting HR is shown in Table 2. Figure 1 is a study example of a patient showing mitral inflow, pulmonary vein inflow and LVOT PW Doppler measurement at baseline AV delay and at optimal AV delay at resting HR of 60 bpm.

**Table 2:** Effect of Biv Pacemaker Optimization on Echo Doppler Variables at Baseline Heart Rate

Echo Doppler Variables	Baseline	Optmial	P Value
**LV Diastolic Filling Time (ms)**	396 ± 75	420 ± 63	0.016

**LVOT VTI (cm)**	17.5 ± 4.8	19.4 ± 5.2	< 0.001

**LV ejection time (ms)**	287 ± 40	298 ± 36	< 0.001

**Pulmonary artery pressure (mm Hg)**	52 ± 7	44 ± 8	0.001

Values are Mean ± SD LV: left ventricle.; LVOT: left ventricular outflow tract; VTI: velocity time integral

### Increased Atrial Pacing

Patients tolerated increasing atrial pacing rate without developing arrhythmia or hypotension. Mean duration of atrial pacing during which Doppler echo data for diastolic filling time at fixed and variable AV delays was collected was 2 minutes. In 13 patients in whom echo Doppler systolic ejection time before and after AV delay optimization was also obtained, mean duration of atrial pacing was 3 minutes.

### Changes in Optimal AV Delay With Increasing Heart Rate

**Figure 2:**
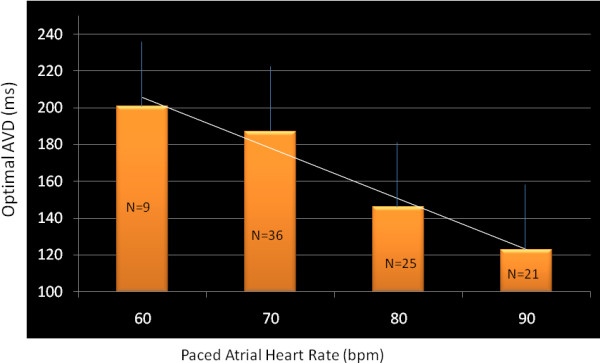
**Effect of increasing paced atrial rate on optimal AV delay in the study population**.

**Figure 3:**
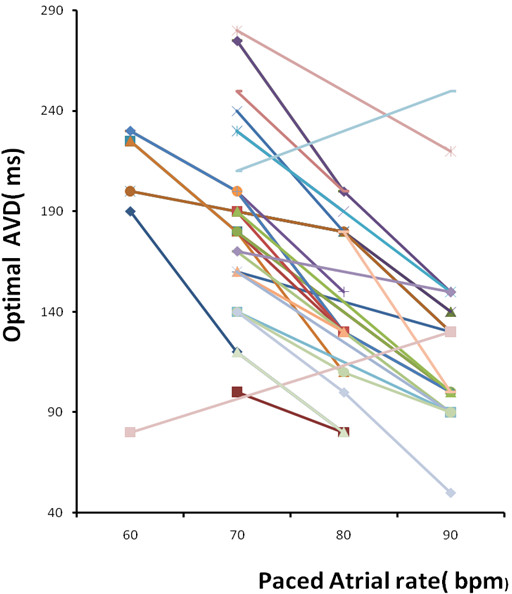
**Optimal AV delay at increased paced atrial heart rates during biventricular pacing in each study patient**.

81 AV delays were evaluated at different atrial pacing rates. Data on optimal AV delay at each paced heart rate in the overall study population is shown in Table 3. In 32 patients, optimal AV delay decreased as atrial paced rate increased. Optimal AV delay was 201 ms at 60 bpm, 187 ms at 70 bpm, 146 ms at 80 bpm and 123 ms at 90 bpm (p = 0.001 by ANOVA for all) and is shown in Figure 2. Repeated measures ANOVA showed a statistically significant shortening of AV delay at three sets of heart rates (60, 70 and 80 bpm, P < 0.001). Optimal AV delay at baseline and increased paced atrial rate in each patient is shown in Figure 3.

**Table 3:** Optimal AV Delay at Different Paced Atrial Rates in the Study Patients

Heart rate (bpm)	N	Optimal AV delay(ms)*	95% Confidence Interval	Minimum AV delay(ms)	Maximum AV delay(ms)
60	9	201 ± 48	169.64-232.36	190	230

70	36	187 ± 44	172.63-201.37	100	280

80	25	146 ± 42	129.54-162.46	80	200

90	21	123 ± 49	102.04-143.96	50	250

Values are Mean ± SD

*Optimal AV delay at different heart rates (P value = 0.001 ANOVA)

### Effect of Optimal AV Delay on Diastolic Parameters

**Figure 4:**
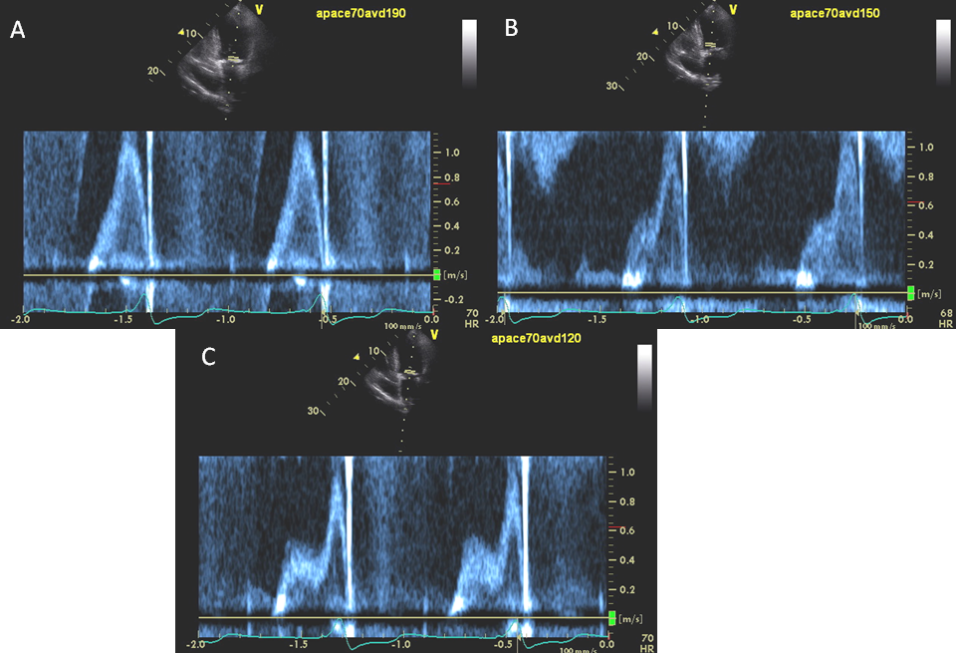
**Increased atrial pacing rate requires progressive shortening of AV delay**. After baseline AV delay optimization shown in Figure 1, atrial pacing rate was increased to 70 beats/min, and AV delay was kept fixed at 190 ms. Marked E and A fusion developed (A). AV delay was then progressively shortened to 120 ms until E and A separation occurred (C). Hence, optimal AV delay at a paced atrial rate of 70 beats/min was 120 ms.

**Figure 5:**
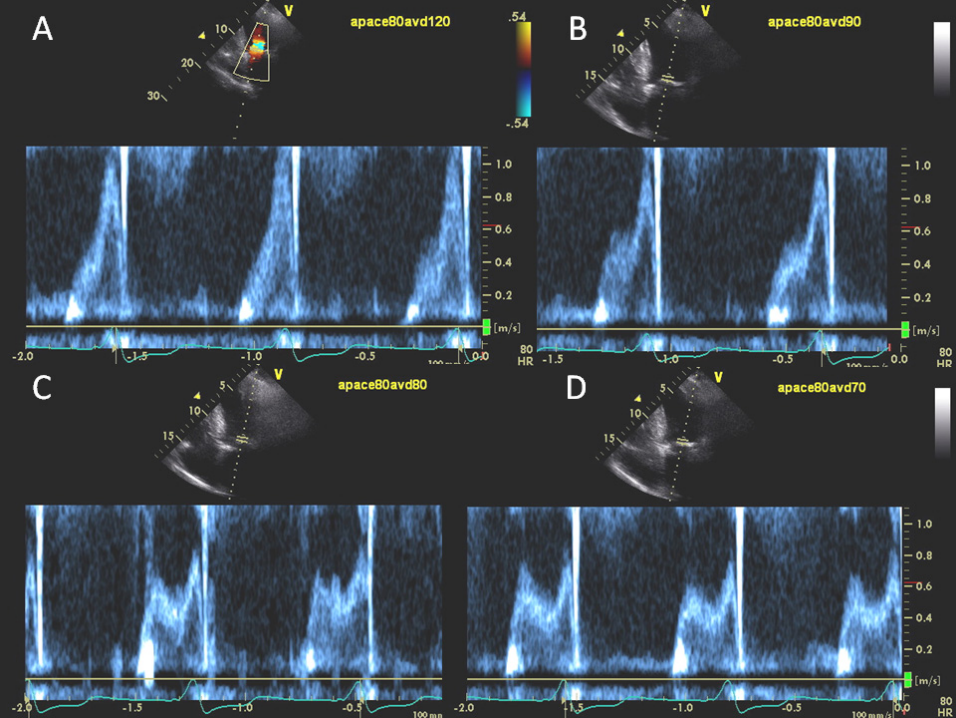
**Increased Atrial Pacing Rate Requires Progressive Shortening of AV delay**. After optimization AV delay at atrial pacing rate of 70 bpm in the same patient as shown in Figure 4, paced atrial rate was then increased to 80 beats/min at the AV delay of 120 ms. Marked E and A fusion developed again (A). Progressive shortening of AV delay to 90 ms (B), and then 80 ms (C) caused mitral inflow E and A separation. Further shortening of AV delay to 70 ms (D) led to shortening of mitral inflow A-wave and premature closure of mitral valve. Hence, optimal AV delay at a paced atrial rate of 80 bpm was 80 ms.

Data on diastolic filling time, mitral inflow VTI, E wave deceleration time, E and A wave velocities, E/A ratio, LVOT ejection time and LVOT VTI at various heart rates during fixed and optimal AV delays is shown in Table 4. Figures 4 and 5 show mitral inflow PW Doppler tracings with increased atrial pacing rate at fixed and optimal AV delay at a heart rate of 70 bpm (Figure 4) and then 80 bpm (Figure 5) in a study patient. Absolute and percent changes in PW Doppler mitral inflow and of LVOT at fixed and optimal AV delay at 81 AV delays tested are shown in Table 5. Diastolic filling time was significantly longer at optimal AV delay with a mean difference at fixed vs. optimal AV delay of 40.3 ± 25 ms, p < 0.001. Decreasing optimal AV delay with increased heart rate was associated with an increase in mitral inflow PW Doppler VTI. No significant change was seen in mitral inflow deceleration time at fixed vs. optimal AV delay. Mitral inflow A wave and E/A ratio showed statistically significant changes after optimization of AV delay at different heart rates (Table 4). LVOT systolic ejection time increased at optimal vs. fixed AV delay at increased heart rates, with a mean change of 11 ± 15 ms (p < 0.02). A trend for an increased in LVOT VTI was noted before and after optimization (Tables 4 and 5).

**Table 4:** Echo Doppler Measurements Before and After Optimization During Atrial Pacing Above Baseline Heart Rate

Echo Doppler Variables	Before Optimization	After Optimization	P value
**Diastolic filling time (ms)**	279 ± 60	318 ± 43	< 0.001

**Mitral inflow VTI (cm)**	14.9 ± 5.7	15.8 ± 4.7	0.04

**E wave (cm/s)**	0.71 ± 0.30	0.72 ± 0.25	0.33

**A wave (cm/s)**	0.70 ± 0.29	0.64 ± 0.26	0.01

**E/A**	1.13 ± 0.51	1.27 ± 0.65	< 0.001

**Systolic ejection time (ms)**	276 ± 37	285 ± 38	0.011

**LVOT VTI (cm)**	14.7 ± 3.8	15.5 ± 3	.21

Values are Mean ± SD. LVOT: left ventricular outflow tract; VTI: velocity time integral

**Table 5:** Effect of Fixed versus Optimal AV Delay on Echo Doppler Parameters at Increased Atrial Pacing Rates

Echo Doppler Variable	Δ Fixed vs. Opt AV Delay	% Δ	P
**Diastolic Filling Time (ms)**	40.2 ± 25	14	< 0.001

**Mitral inflow VTI (cm)**	0.92 ± 2	5	0.04

**Systolic Ejection Time (ms, n = 13)**	11 ± 15	4	0.011

**LVOT VTI (cm, n = 13)**	0.56 ± 1.6	3	0.21

Values are Mean ± SD or percent change

### Final Pacemaker Settings

Rate adaptive AV delay feature was turned ON in all patients and AV delay shortening was programmed according to AV delays found optimal at increased paced heart rate in each patient. In patients with St Jude device, AV delay settings were changed to the "most aggressive" AV delay shortening.

25 of the patients reported an improvement in their ability to walk immediately after optimization compared to before optimization and 5 patients were unable to tell the difference. No patient reported deterioration in exercise tolerance. NYHA improved from 2.5 ± 0.8 at baseline to 1.7 ± 0.8 (p < 0.01) at one month post optimization. MHFQA Failure Questionnaire was returned at the end of one month by 20 subjects. MHFQA improved from 49 ± 30 to 39 ± 30 (p = 0.2). When emotional and physical components of assessment were separated, a significant improvement was noted in the physical component of quality of life from 32 ± 17 to 25 ± 12, p < 0.05.

### NYHA Class by Device Manufacturer

There were 14 Medtronic devices, 7 Boston Scientific devices and 10 St. Jude devices. Mean NYHA improvement for St Jude was 0.10 compared to 0.87 for all other brands (p = 0.068). No differences were found for other brands.

## Discussion

The salient findings of our study are that increased atrial pacing rate causes a marked shortening of diastolic filling time at a fixed AV delay and a shortening of AV delay at increased heart rate improves diastolic filling. This shortening of AV delay is associated with an improvement in LV ejection time and indirectly LV stroke volume. Optimization of AV delay at rest and programming of aggressive rate adaptive delay was associated with an improvement in NYHA functional class at 1 month follow up. The shortening of AV delay at increased heart rates allows adequate diastolic filling time by causing separation of early and late diastolic filling and in turn leads to an improvement in cardiac ejection as measured by LV systolic ejection time. Aggressive shortening of AV delay at increased heart rates is further supported by the improvement in functional class that occurred in our patients at one month follow up.

Modern pacemaker devices allow rate-response as well as rate-adaptive AV delay shortening during exercise. Current pacemakers do not allow AV delay lengthening at increased heart rates. In our study the amount of AV delay shortening needed to optimize diastolic filling was more aggressive than current default automated pacemaker algorithms provide. This could be related to current rate-adaptive AV delay algorithms based on data derived from patients with conduction abnormalities who had otherwise preserved right ventricular and LV systolic function whereas patients with CRT have marked cardiac dysfunction.

We did not have a control group in whom rate adaptive AV delay shortening was not performed to differentiate effects of optimization at resting heart rates vs. optimization at resting heart rates and use of rate adaptive AV delay, however improvement in NYHA class and quality of life in our patients suggests that programming of aggressive rate adaptive delay shortening is not deleterious, as some studies suggest, and is likely to be beneficial. We selected patients who were atrial pacing in addition to Biv pacing at baseline since a constant heart rate provided by atrial pacing allowed patients to act as their own control in whom fixed versus shortened AV delay effects in a given cardiac cycle of constant length could be tested.

In humans with normal cardiac function, there is decreasing AV delay with increasing heart rate in a predictable manner (4 ms/10 bpm). Patients with dilated cardiomyopathy have prolongation of LV systole and an abnormal shortening of LV diastole at rest [13] that is accentuated during exercise [11]. In addition, abnormal shortening of LV diastolic time requires higher left atrial pressures to maintain LV filling during exercise resulting in pulmonary congestion and exercise intolerance [10,12]. Although previous studies showed the importance of periodically optimizing AV delay [14] there is insufficient data on changes in AV delay with pacing at various heart rates. These studies are summarized in Table 6. Scharf et al [8] attributed probable longer diastolic filling with prolonging of AV delay that caused an improvement in LVOT VTI. However physiologically, prolongation of AV delay should lead to approximation of E and A waves due to pushing back of atrial contraction towards early diastole when early diastolic filling (E wave on PW Doppler) occurs. Calculation of AV delay immediately after exercise with unstable heart rates may have confounded their results.

**Table 6:** Prior Studies That Evaluated the Effect of Increased Heart Rate on Optimal Atrioventricular Delay in Patients with CRT

Author	N	Study Population	Heart rate tested	How HR Increased	Method to calculate Opt AV Delay	Optimal AV Delay at Increased Heart Rate
Melzer et al	20	CRTresponder 64 ± 10 yrs, NYHA < 3. VDD and DDD mode used. EF 23.2 ± 7.6	22.5 ± 9.6 bpm above baseline Supine bicycle ergometer beginning with 25 W and increasing the workload by 25 W every 2 min. (71+9 W).	Supine bicycle exercise	Combination of mitral inflow PW Doppler, trans-esophagel left atrial electrograms and surface ECG.	No AV delay change needed in VDD mode. With DDD mode, optimal AV delay was shortened by 2.6 ms/10 bpm

Scharf et al	36	Biv-ICD, 62 ± 8 yrs, EF 17 ± 5	Pacing heart rate increased to 110-120 bpm and with exercise increase in intrinsic heart rate at least 20 bpm above baseline. Optimal AV Delay determined post exercise	DDD pacing or treadmill exercise In 22 patients with DDD HR increased to 110-120 bpm. In 14 patients with exercise HR increased at least 20 bpm above baseline	LVOT VTI post exercise	Prolongation of AV delay found optimal at increased HRs. An increase in LVOT VTI of 0.047 cm/s per 20 ms prolongation of AV delay per 10 bpm increase in heart rate for DDD pacing and 0.146 cm/s increase in VTI per 20 ms prolongation of AV delay per 10 bpm increase in heart rate during exercise. Beneficial effect of AV delay prolonging was observed until heart rate 110 bpm.

Grimm R et al	15	CRT patients without atrial pacing who were able to exercise, 57 ± 16 yrs, EF 37 ± 15	Atrial-sensed Biv pacing, HR 20-40 bpm above baseline	Supine bicycle exercise	Maximum LV filling time. The duration of LV filling, stroke volume, and a clinical assessment of LV function were studied.	AV delay shortening needed at increased HR for all patients using three independent criteria. Consistent trends were observed between all three parameters for 12 out of the 15 patients.

Mokrani B et al	50	CRT patients who were able to exercise, 69 ± 7 yrs, EF 25 ± 7	Atrial-sensed Biv pacing, 60% of the maximal predicted. HR, with the sensed AV delay set at 40, 70, 100, 120, 150, and 200 ms. Only 1 maximum HR tested.	Supine bicycle exercise	LVOT VTI, LV filling time	Optimal AV delay based on LVOT-VTI was shorter during exercise than at rest in 37%, unchanged in 37%, and longer in 26% of patients. The optimal AV delay based on mitral inflow filling time was shorter during exercise than at rest in 27%, unchanged in 23%, and longer in 50% of patients. Opt-imization of AV delay during exercise inc-reased LV filling time and LVOT-VTI (P < .05)

Valzania C et al	24	CRT patients able to exercise, 63 ± 9 yrs, EF 36 ± 9	Atrial-sensed Biv pacing, 20-beat increase in HR above baseline. Only 1 maximum HR tested.	Supine bicycle exercise	LVOT VTI by PW Doppler and automated intra-cardiac electrogram (IEGM)to optimize AV delay and VV delay	Optimal VV delay varied considerably from rest to exercise, while AV delay did not change. A substantial agreement in deriving optimized AV delays was observed between the echocardiogram and the IEGM method, both at rest and during exercise.

Whinnett ZI et al	20	CRT patients who were able to exercise, 68 yrs(46-82 yrs)	Atrial-sensed Biv pacing, HR of 100 bpm with exercise. Pacing at rest at 5 bpm above resting rate and at 100 bpm. Sensed-paced difference, calculated as an "expected" value for the exercise optimum.	Treadmill exercise	Noninvasive finger arterial pressure measurements using a Finometer	Hemodynamic optimization of AV delay under three different conditions before exercise. The resting three-phase model correlated well with the actual exercise optimal AV delay (r = 0.85, mean difference ± standard [SD] = 3.7 ± 17 ms). In 11 patients, the optimal AV delay was shorter during exercise than at rest, in eight patients it was longer and in 1 patient, unchanged.

Tse Hung-Fat et al	20	CRT patients who were able to exercise, 65 ± 4 yrs, EF 27 ± 3	AV delay adaptive algorithm, maximum programmed equal to the optimal resting AV delay during atrial pacing and the minimum AV delay to the optimal resting AV delay during atrial pacing--50 bpm in 10-ms decrements.	Cardiopulmonary treadmill exercise	Longest LV filling time without truncation of the A wave from mitral inflow PW Doppler	In heart failure patients with severe chronotropic incompetence as defined by failure to achieve < 70% target HR, appropriate use of rate-adaptive pacing with CRT provides incremental benefit on exercise capacity during exercise.

Recent study by Mokrani et al [15] found that variable changes in optimal AV delay were needed during exercise when using LVOT VTI and diastolic filling time in patients. Changing heart rates during Doppler assessment may have led to variable results. Study by Valzania et al found variation in VV delay from rest to exercise but no significant changes in AV delay using LVOT VTI to optimize AV delay during rest and exercise [16].

Contrary to findings of these earlier studies, several other studies found that shortening of AV delay is needed at increased heart rates. Melzer et al [9] found that optimal AV delay was shorter with increasing heart rate during DDD (atrial-paced Biv pacing) pacing and not during VDD (atrial-sensed Biv-pacing) pacing during exercise. All patients included in this study were responders by NYHA class and were enrolled 6 months post CRT. To calculate optimal AV delay, interatrial conduction time (time between atrial stimulus and left atrial deflection), left atrial electromechanical time (time between left atrial deflection and the end of the A wave on mitral diastolic filling) and LV latency period (time between ventricular pacing stimulation and end of truncated A wave on programming the pacemaker at a non-physiologic short AV delay) were measured. During exercise, interatrial conduction time and LV latency period did not change significantly but left atrial electromechanical action decreased significantly with patients in the DDD mode. Thus stable electrical conduction time but variable diastolic filling time at different heart rates was found in this study.

Whinnett et al [17] determined optimal AV delay at rest and during exercise using non invasive finger arterial pressure measurements. Their results showed shortening of AV delay at increased heart rate in 11 patients while 8 patients needed lengthening of AV delay. Measurements of optimal AV delay at three different phases as described above at rest could predict the exercise optimal AV delay with good agreement (r = 0.85), however only using optimal AV delay during atrial sensing at rest was less reliable in predicting optimal AV delay at increased heart rates. The study showed predictive value of atrial pacing at rest for optimal exercise AV delay.

Tse et al [18] evaluated changes in AV delay in CRT patients during exercise treadmill test using mitral inflow PW Doppler parameters. AV delay optimization by echocardiography provided the longest mitral filling time without truncation of A wave. 55% of patients had severe chronotropic incompetence and achieved < 70% age-predicted heart rate during exercise. These patients showed increased peak exercise heart rate, exercise time and peak oxygen consumption by appropriate use of rate-adaptive pacing.

Our study differs from these earlier studies in that our study population is older, on average by 10 years, had a higher NYHA class, most were unable to perform more than mild exercise and all were DDD pacing indicating conduction system disease. In addition ours is the only study which was not only observational but rate adaptive AV delay was programmed according to findings of optimal AV delay and increased atrial pacing rate. Another differentiating feature of our study that 32 of our 34 patients required a decrease in AV delay with increasing heart rate and the amount of AV shortening was more aggressive than found by previous studies. We used atrial pacing at incremental heart rates in the physiologic range to mimic heart rates expected to be achieved in these patients during physiologic exercise. Our study also differs from above studies that we used mitral inflow and LVOT Doppler as well as pulmonary vein Doppler echocardiography to determine optimal AV delay at resting heart rates. In addition we showed in a subset of our study patient's improved systolic ejection time at optimal shortened versus fixed AV delays at increased heart rates. Above all we showed improvement in functional class as a result of pacemaker changes including use of dynamic rate adaptive AV delay shortening as determined by our pacing protocol.

The differences in NYHA class improvement show a trend to lower improvement in patients with St Jude device. This is likely due to a different AV delay shortening algorithm in St Jude devices that does not allow AV delay shortening until a heart rate of 90 bpm and at that heart rate is programmable at 1-, 2-, or 3-ms shortening per 1 beat/min in rate (low, normal, high) vs. linear response between shortest heart rate and maximum heart rate for other devices. Given no other significant differences between manufacturer algorithms, this suggests that rate adaptive AV delay shortening is an important element in pacemaker programming.

Our finding suggest that individualized pacemaker optimization should be performed at rest and at increased heart rates and more aggressive shortening of the AV delay should be used as default pacemaker setting in most devices in patients with CRT who are in the atrial pacing Biv pacing mode (DDD pacing).

## Limitations

Atrial pacing that may be less physiologic than intrinsic increase in atrial rate during exercise hence our findings may not apply to patients with predominant atrial sensing. Our use of dynamic AV delay shortening as determined by atrial pacing was limited by device algorithms by different manufacturers. We did not perform invasive study to assess stroke volume changes with various AV delay at different heart rates.

We evaluated functional class by NYHA and quality of life questionnaire and did not perform follow up echocardiographic assessment. However we do not anticipate a significant change in LVEF over time given prolonged time period since pacemaker implant. While improvement in functional class may have been subjective, it likely was a result of pacemaker optimization since no changes occurred in medications or medical or cardiac condition in our patients. Finally our study is not randomized that would allow true impact of rate adaptive AV delay change. Such a study should be performed.

## Conclusion

Our data suggests that an individualized pacemaker programming should be performed. In most patients with CRT an increase in heart rate causes a compromise in diastolic filling which can be improved with a significant shortening of AV delay at increased pacing rates. Aggressive AV delay shortening as determined by acute atrial pacing protocol was associated with a significant improvement in functional class post CRT.

## Competing interests

The authors declare that they have no competing interests.

## Authors' contributions

RR participated in measuring echo data and writing the manuscript. SQ and NT collected the information, followed patient and carried out entering data. AO performed statistical analysis. TZN conceived of the study, designed and coordinated each step and revised the final version of manuscript prior to submission. All authors read and approve the final manuscript.
